# Health financing policies during the COVID-19 pandemic and implications for universal health care: a case study of 15 countries

**DOI:** 10.1016/S2214-109X(23)00448-5

**Published:** 2023-11-14

**Authors:** Chuan De Foo, Monica Verma, Si Ying Tan, Jess Hamer, Nina van der Mark, Aungsumalee Pholpark, Piya Hanvoravongchai, Paul Li Jen Cheh, Tiara Marthias, Yodi Mahendradhata, Likke Prawidya Putri, Firdaus Hafidz, Kim Bao Giang, Thi Hong Hanh Khuc, Hoang Van Minh, Shishi Wu, Cinthya G Caamal-Olvera, Gorka Orive, Hong Wang, Stefan Nachuk, Jeremy Lim, Valeria de Oliveira Cruz, Rob Yates, Helena Legido-Quigley

**Affiliations:** a Saw Swee Hock School of Public Health, National University of Singapore, Singapore; b Duke NUS Graduate Medical School, Singapore; c Centre for Universal Health, Chatham House, London, UK; d Faculty of Social Sciences and Humanities, Mahidol University, Nakhon Pathom, Thailand; e National Health Foundation, Bangkok, Thailand; f Faculty of Medicine, Chulalongkorn University, Bangkok, Thailand; g Department of Health Policy and Management, Faculty of Medicine, Public Health, and Nursing, Universitas Gadjah Mada, Yogyakarta, Indonesia; h Nossal Institute for Global Health, The University of Melbourne, Melbourne, VIC, Australia; i School of Preventive Medicine and Public Health, Hanoi Medical University, Hanoi, Viet Nam; j Hanoi University of Public Health, Hanoi, Viet Nam; k Dalla Lana School of Public Health, University of Toronto, Toronto, ON, Canada; l Universidad Autónoma de Nuevo León, Monterrey, Mexico; m NanoBioCel Group, Laboratory of Pharmaceutics, School of Pharmacy, University of the Basque Country UPV/EHU, Vitoria-Gasteiz, Spain; n Bill & Melinda Gates Foundation, Seattle, WA, USA; o South-East Asia Regional Office, WHO, New Delhi, India; p Imperial College and the George Institute for Global Health, London, UK

## Abstract

**Background:**

The COVID-19 pandemic was a health emergency requiring rapid fiscal resource mobilisation to support national responses. The use of effective health financing mechanisms and policies, or lack thereof, affected the impact of the pandemic on the population, particularly vulnerable groups and individuals. We provide an overview and illustrative examples of health financing policies adopted in 15 countries during the pandemic, develop a framework for resilient health financing, and use this pandemic to argue a case to move towards universal health coverage (UHC).

**Methods:**

In this case study, we examined the national health financing policy responses of 15 countries, which were purposefully selected countries to represent all WHO regions and have a range of income levels, UHC index scores, and health system typologies. We did a systematic literature review of peer-reviewed articles, policy documents, technical reports, and publicly available data on policy measures undertaken in response to the pandemic and complemented the data obtained with 61 in-depth interviews with health systems and health financing experts. We did a thematic analysis of our data and organised key themes into a conceptual framework for resilient health financing.

**Findings:**

Resilient health financing for health emergencies is characterised by two main phases: (1) absorb and recover, where health systems are required to absorb the initial and subsequent shocks brought about by the pandemic and restabilise from them; and (2) sustain, where health systems need to expand and maintain fiscal space for health to move towards UHC while building on resilient health financing structures that can better prepare health systems for future health emergencies. We observed that five key financing policies were implemented across the countries—namely, use of extra-budgetary funds for a swift initial response, repurposing of existing funds, efficient fund disbursement mechanisms to ensure rapid channelisation to the intended personnel and general population, mobilisation of the private sector to mitigate the gaps in public settings, and expansion of service coverage to enhance the protection of vulnerable groups. Accountability and monitoring are needed at every stage to ensure efficient and accountable movement and use of funds, which can be achieved through strong governance and coordination, information technology, and community engagement.

**Interpretation:**

Our findings suggest that health systems need to leverage the COVID-19 pandemic as a window of opportunity to make health financing policies robust and need to politically commit to public financing mechanisms that work to prepare for future emergencies and as a lever for UHC.

**Funding:**

Bill & Melinda Gates Foundation.

## Introduction

4 years since the start of the COVID-19 pandemic, governments everywhere continue to grapple with the consequences of the pandemic and the consequences resulting from their national responses to it. With an estimated 771 million cumulative confirmed cases of COVID-19 and 6·96 million deaths as of Oct 12, 2023, and with more than 70 million people pushed into poverty by the end of 2020 because of the pandemic,^1^ this crisis can potentially derail progress towards achieving the Sustainable Development Goals (SDGs). Yet, not all countries have been affected to the same extent, even among those with similar income levels, in part as a result of the policy choices they made.^2^ These choices were shaped and constrained by flexibility to expand funding as countries adopted economic and social measures to mitigate the impact of the pandemic. Notably, US$16 trillion was disbursed globally through fiscal packages as of March, 2021, with high-income countries mobilising an equivalent of more than 16% of their gross domestic products (GDPs) and low-income countries mobilising 1·6% of their GDPs.^3^ Countries that entered the pandemic without robust health financing mechanisms have had challenges with the rapid deployment of funds for their national COVID-19 responses.^4^

**Research in context**

**Evidence before this research**

Across the world, countries have adopted diverse approaches to finance their COVID-19 responses. However, most studies only looked at the COVID-19 responses at the health system level in its entirety and how universal health coverage (UHC) was a facilitator to provide health-care services to the population during the pandemic. In fact, most studies examining health-care financing policies focused on strengthening health financing systems in general, and did not lie within the ambit of pandemic response financing. From a UHC perspective, most studies explored health financing reforms for one or a group of countries, often devoid of elements of health emergency readiness. We identified two studies that bore semblance to our own. However, these two studies were region specific, namely in southeast Asia and Europe, whereby the authors explored health financing policies during the COVID-19 pandemic. Therefore, there is a gap in the literature on how the spectrum of health financing policies deployed at the global level influences countries’ national responses and how the financing landscape needs to be reformed to encompass resilient financing strategies while moving towards UHC. We searched PubMed, EMBASE, Scopus, and Google Scholar between Dec 19, 2021, and Oct 30, 2022, and updated the search on May 26, 2023, for studies investigating how health systems financed their national responses during the COVID-19 pandemic. We used the following search terms: for PubMed, (“Financing”[MeSH] OR “Financial Management”[MeSH] OR “Financing Government”[MeSH] OR “Health Expenditure”[MeSH] OR “Health Insurance”[MeSH] OR “User Fee”[MeSH] OR “Innovative Financing”[MeSH] OR “Payment Mechanism”[MeSH]) AND (“Healthcare” OR “Health system” OR COVID*); for EMBASE, (“health financing”/exp OR “health expenditure”/exp OR “financing government”/exp OR “health insurance”/exp OR “payment mechanism”) AND (“healthcare”/exp OR “health system”/exp OR “COVID”/exp); for Scopus, TITLE-ABS-KEY (financing OR financial management OR financing government OR health expenditure OR health insurance OR user fee OR innovative financing OR “payment AND mechanism” OR “health AND budget” OR “health AND insurance”) AND (“healthcare” OR “health AND system” OR covid-19 OR “pandemic”); and for Google Scholar, we used the aforementioned MeSH terms and sieved through the first ten pages of the searches because of the heterogeneity and amount of results yielded through this platform. Our search was not restricted to study type or language.

**Added value of this study**

We provide robust evidence of health-care financing policies deployed by 15 countries representing all WHO regions and offer an overview and illustrative examples of the modifications made to these policies for countries to rapidly mobilise fiscal resources for health. We also found that countries should constantly plan and assess their health financing policies and iteratively modify them to meet the changing needs of the population and evolving economic situation affected by non-domestic factors. Accountability and monitoring were central in ensuring fiscal resources reached targeted entities, personnel, and populations, which should be upheld by strong governance and coordination, information technology, and community engagement principles. We organised key themes from our data into an evidence-informed framework for resilient health financing.

**Implications of all the available evidence**

Countries can be better prepared for future health crises by committing to resilient health financing mechanisms that enable their health systems to expeditiously mobilise fiscal resources, combined with efficient and effective use of new funds, to respond to shocks and recover from them. Now more than ever, health systems globally need to leverage the COVID-19 pandemic as a window of opportunity to make health financing structures more resilient and move countries towards UHC.

The pandemic foregrounded structural and social issues whereby vulnerable groups were disproportionately affected by the loss of access to health care and other essential services, thereby exacerbating existing inequalities. A constrained fiscal space coupled with competing national needs makes balancing spending on health and the wider social economic support to vulnerable groups a difficult balancing act.^5^ Consequently, a medium and long-term impact on population health and the economy should be expected.^6^, ^7^ Furthermore, fiscal space for health is increasingly threatened in the face of a lingering economic recovery because of the pandemic compounded by global supply chain disruptions and other global conflicts. Increasing interest payments on public debt together with increased interest rates to limit inflation further threaten countries’ capacity to invest in health.

The pandemic also had a disproportionate impact on front-line health-care workers, leading to workforce shortages worldwide. In settings with pre-existing scarcity of health-care workers, the burden fell on already stretched and understaffed health systems. As of October, 2022, more than 115 000 health workers were estimated to have died of COVID-19 contracted in hospitals, about a third of all health workers reported to have anxiety and depression, and nearly half had burnout.^8^, ^9^ A range of interventions were adopted to support, retain, and motivative the health-care workforce during the pandemic, including retention bonuses and other financial incentives. To that end, appropriate funding also had to be mobilised to mitigate this challenge. Considering these aspects, prioritisation of workforce strengthening and planning is thus crucial to avoid another public health crisis caused by structural challenges.

These observations highlight the inadequacy of existing health financing systems and call for sustainable and effective capacities for mobilising financial resources to strengthen health systems in the context of a health crisis and global economic uncertainty. Going forward, resilience in health financing systems will be foundational for recovery, which encompasses reinvigorating all dimensions of the health system to prepare for and prevent future health threats. A resilient health system can absorb external shocks and rebound stronger but requires an equally resilient financial ecosystem. This Article operationally defines resilient health financing as the ability and capacity to prepare for, manage, recover, and build back better from shocks brought on the health system through fiscal mechanisms that protect the lives and livelihoods of populations. The key elements of a resilient health financing strategy are universal coverage, with additional protection for disadvantaged groups; a predominant reliance on public revenues to ensure that out-of-pocket expenditure is low; and sufficient and flexible public financing. These elements are relevant to the key pillars of universal health coverage (UHC).^10^ Unfortunately, the COVID-19 pandemic has affected the progress towards UHC made in the past two decades, having contributed to the worst economic crisis since the 1930s.^11^ Notably, UHC, when successfully pursued, makes health service delivery and financing models more sustainable and equitable while supporting health services, crucial in health emergencies, to ensure that everyone, especially vulnerable individuals, receives adequate care.^12^

Understanding the processes of resource mobilisation, the magnitude of resources, and the mechanisms of funding disbursement is paramount to comprehend the context of fiscal policies deployed and prepare for any future crisis. Previous studies have assessed health financing measures during the peak of the COVID-19 pandemic in several countries, including Pakistan, Nigeria, and Romania.^13^, ^14^, ^15^ Other studies have documented challenges and prospects for developing national health financing systems against the backdrop of falling GDPs.^16^ Previous research assessed the financial effect of the pandemic on health-care providers in England (UK), Germany, Israel, and the USA,^17^ underscored the need for sustainable health financing to steer through the COVID-19 crisis in Asia,^18^ and documented adjustments made to hospital payment systems in Europe.^19^ More specifically, Barasa and colleagues^20^ outlined a research agenda to enable countries to mitigate the health financing challenges and emerge with more resilient health financing systems. At the regional levels, studies have reviewed the health financing policy measures adopted in southeast Asia^21^ and Europe,^10^ but systematic documentation of fiscal policies at the global level and with countries of varying UHC indices remains absent. Therefore, in this Article, we collate evidence on health financing strategies adopted during the pandemic while placing resilience at the core of emergency preparedness. We build a clear case for resilient health financing to be integrated within the concept of UHC. As such, we aim to describe health financing policies adopted in 15 countries during the pandemic; develop a conceptual framework for resilient health financing; and argue a case for using the pandemic as a window of opportunity to advance towards UHC.

## Methods

### Country selection

We examine the health financing policy responses to the COVID-19 pandemic through case studies in 15 countries, purposefully selected to include a range of income levels, UHC index scores, and health system typologies (table). We ensured that all WHO regions were represented.

**Table:** Countries reviewed and their indicators

	**Confirmed COVID-19 deaths as of May 26, 2023**	Population vaccinated with the initial vaccination protocol as of March 31, 2023*	**Current health expenditure in 2020 (% of GDP)**^22^	**Out-of-pocket expenditure in 2020 (% of current health expenditure)**^23^	**UHC service coverage index 2019**
Brazil	704 659	81·81%	10·31%	22·39 %	75
China	121 714	89·54%	5·59%	34·79%	82
Germany	174 979	76·24%	12·82%	12·54%	86
India	532 031	67·17%	2·96%	50·59%	61
Indonesia	161 918	62·68%	3·41%	31·79%	59
Iran	146 386	66·11%	5·34%	37·06%	77
Mexico	334 586	64·19%	6·24%	38·77%	74
Nigeria	3155	31·94%	3·38%	74·68%	44
Singapore	1872	90·85%	6·05%	18·97%	86
Spain	121 852	85·65%	10·71%	19·62%	86
South Africa	102 595	35·13%	8·58%	5·36%	67
Thailand	34 473	74·59%	4·36%	10·54%	83
UK	229 307	75·19%	11·98%	13·60	88
USA	1 127 152	69·41%	18·82%	9·89%	83
Viet Nam	43 206	87·54%	4·68%	39·60%	70

Data are n, unless otherwise stated. Data were taken from Our World in Data, unless otherwise referenced. GDP=gross domestic product. UHC=universal health coverage.

* Initial vaccination protocol refers to the receipt of two doses for most vaccines and one dose or three doses for a few other vaccines.

### Study design

We used a four-step process to explore the national health financing policy responses implemented during the pandemic (appendix p 1). The initial data gathering was complemented with semi-structured, in-depth interviews with 61 key stakeholders in the fields of health systems and health financing. After validating the data, key themes were organised into a conceptual framework for resilient health financing for health emergencies, by adopting a combination of inductive and deductive approaches. The National University of Singapore Ethics Committee approved this study (NUS-IRB-2021-172). QSR NVivo (version 12) was used to do the thematic analysis.^24^ Full details of the methods are elaborated in the appendix (pp 2–3).

### Role of the funding source

The funder of the study had no role in study design, data collection, data analysis, data interpretation, or writing of the report.

## Results

We identified two key phases with regard to resilient health financing: (1) absorb and recover, where countries absorb the initial shock to health systems and establish mechanisms to recover from it, tackling the almost inevitable backlog of usual care (panel 1); and (2) sustain, ensuring that earlier progress towards sustainable health financing is maintained, and advancement and commitment to UHC remain priorities (panel 2). The absorb and recover phases were merged because they involve similar processes (figure).

Panel 1 **Examples of financing policies used during the absorb and recover phases of the COVID-19 pandemic**

**Extra-budgetary funds**

*National reserves mobilisation*

In Brazil, the COVID-19 response was primarily financed from Brazilian National Treasury Bonds, funds approved under four Emergency Constitutional Amendments that bypassed conventional budget regulations, and the Unified Health System, for pandemic-related fiscal support.

In Germany, €10·5 billion (US$11·5 billion) were mobilised from the liquidity reserve of the Central Reallocation Pool (German Health Fund), until September, 2020, to compensate for beds reserved for patients with COVID-19, reduce occupancy rates, and expand intensive care units to free up hospital capacity in case of sudden surges.

In Indonesia, the central government reserves were mobilised to rapidly obtain financial resources to augment the country's national response in the earlier stages of the pandemic.

In Iran, the Government drew from national reserves (comprising various domestic taxes, both direct and indirect, oil revenues, and the sale of nationally owned shares of companies) to combat the pandemic, particularly during the earlier stages.

In Singapore, the President authorised the use of S$31 billion (US$23 billion) of national reserves in June, 2020, for job support, health system strengthening, and economic stabilisation.

*Loans taken up by governments*

In India, the World Bank approved US$2·75 billion in emergency lending between March, 2020, and June, 2020, of which $2·25 billion was disbursed by December, 2020. $1 billion was committed for health support (COVID-19 Emergency Response and Health System Preparedness Project), $1·15 billion for social protection, and $750 million for small business support.

In Nigeria, in April, 2020, the Finance Minister stated Nigeria would seek US$6·9 billion from multilateral lenders for food support, vaccine procurement, and mitigation of COVID-19 impact on HIV, tuberculosis, and malaria programmes. The country obtained $3·4 billion in emergency support from the International Monetary Fund to address the COVID-19 pandemic. Additionally, the World Bank approved a package of $1·5 billion in December, 2020, to build a resilient recovery after the pandemic, and the African Development Bank gave a loan of $289 million under the COVID-19 Rapid Response Facility—Nigeria's request was submitted in March, 2020, and it was approved in June, 2020.

In South Africa, a US$1 billion emergency Programme Loan from the New Development Bank was approved for South Africa's economic recovery from COVID-19 in April, 2021, which focused on creating new employment opportunities.

In Thailand, the Government raised US$49 billion via two Emergency Loan Decrees in April, 2020, and in May, 2021, of which $9·5 billion was used for public health responses.

*Donations received by governments*

In Indonesia, at the beginning of the pandemic, one of Indonesia's largest coal producers, Adaro Energy, had offered the Government close to US$1 million for the country's national response, whereby the bulk was allocated for logistical support and medicines to protect health-care workers.

In Iran, Japan's Government donated US$6·3 million to Iran in October, 2021, to facilitate the country's immediate response to the pandemic and bolster health capacity in the longer term.

In South Africa, the US Government contributed US$54 million to the country's COVID-19 response, of which USAID's contribution was $17·9 million, to help fund the distribution of vaccines, including the development of field hospitals and surveillance data to monitor progress.

In Viet Nam, the USA donated US$24 million in COVID-19 assistance in March, 2022, to expand COVID-19 facilities and promote equitable access to COVID-19 vaccines.

**Repurposing of existing funds**

*Inter-budget reallocation*

In India, funds were drawn from the State Disaster Response Fund, state contingency funds and through the Post Devolution Revenue Deficit Grant to augment the COVID-19 emergency response package.

In Indonesia, several regulations from the Ministry of Finance were modified to permit national funds for use on public health measures (by redistributing some of the line items to pandemic-related efforts) as well as authorising the use of Village Funds for COVID-19 programmes.

In Mexico, the Health Fund for Wellness, designed to be more flexible, and operated as a trust fund independent of federal budget allocations by the Secretariat of Finance, was used to procure medical supplies quickly for the population.

In South Africa, funds were redirected from existing programmes, multilateral loans, and surplus funds from institutions (eg, the Unemployment Insurance Fund), and repurposed for the country's pandemic response.

In Thailand, the 2020 fiscal budget was reallocated from all ministries by lowering at least 10% of the total budget of each ministry to pool at the central budget level to fund the national response. The Royal Thai Navy cut its 2020 budget by 33%, or US$125 million, to be used for the COVID-19 response.

*Earmarking of funds for pandemic response*

In Brazil, the Federal Government passed a package of about 8·6% of the country's gross domestic product (GDP) for pandemic measures. For states and municipalities, an amount worth R$60 billion (US$11 billion), or 0·9% of GDP, was earmarked to compensate for local tax revenue loss and related financial expenses.

In Germany, €6 billion (US$6·5 billion) was earmarked for the Federal Ministry of Health from the federal budget to support measures such as free rapid testing.

In Mexico, the Mexican Senate voted on October, 2020, to pass a bill to cut funding from 109 public trust funds, into which the Government pays Mex$3 billion (US$145 million), which has been earmarked for pandemic response.

In Spain, a contingency fund, integrated into the national budget to finance urgent needs, was earmarked for the Ministry of Health. Additional funds could be mobilised irrespective of the usual restrictions on public debt and deficit control.

In Thailand, the Emergency Loan Decrees earmarked for medical and public health responses were used for various dimensions, such as risk compensation for public health staff, buying of medical supplies, and treatment and vaccine procurement.

**Fund disbursement mechanisms**

*Protocols or regulations that quicken the disbursement of funds*

In China, insurance companies established emergency response teams to closely contact hospitals and other cooperative units to facilitate fast-track lanes to provide claim settlement services for patients.

In Spain, the Government centralised its procurement efforts and selectively relaxed the criteria for entering administrative agreements related to COVID-19 services, thereby removing the previous authorisation from the Ministry of Finance and legal service, while agreements take effect upon signing instead of post-registration in the Official Gazette.

In India, bureaucratic requirements for the purchase of medical supplies were streamlined, and authorising ministries could charge emergency spending to a special budget up to a specific limit without the approval of the Ministry of Finance, while the decentralisation of budget allocations to line ministries was also permitted.

In Thailand, the National Health Security Office shortened the process of benefit approval for COVID-19-related benefits via the green channel for benefits consideration related to the pandemic.

In the UK, contracting deadlines and sanctions for contracting and payment procedures between the National Health Service (NHS) Commissioners and Foundation Trusts were relaxed, expediting payments on a block-booking basis for new contracts (for private providers and NHS Trusts) and bringing forward scheduled payments made on predicted annual contract values.

In Viet Nam, units in the health system only needed to make an estimate, and the Ministry of Health delivered the estimated quantum without going through the appraisal and approval process to quicken budget adjustments and disbursement.

*Digital technology in aiding funds disbursement*

In Brazil, Bolsa Familia, a cash transfer programme where beneficiaries received the payment through their bank account or debit card, was leveraged for funds transfer to the population. The lump-sum amount was transferred via a state-owned commercial bank, which distributed the funds transferred by the Federal Government, including COVID-19 aid packages.

In India, the Public Financial Management System was used for some insurance premium payments and relief measures, leveraging the system's existing database. This system was also used for direct payments to beneficiaries of social support packages through a direct benefit transfer scheme.

In Indonesia, at the subnational level, several provinces and districts had established either a dashboard of funding allocation or publicly accessible budget documents, which, however, contained limited information and were not uniform across regions.

In Nigeria, the Rapid Response Register was set up as a temporary additional social grant in addition to the National and State Social Registers for urban and peri-urban households impoverished due to the COVID-19 pandemic. The scheme enrolled people through SMS registration to facilitate cash transfers.

In the USA, Economic Impact Payments paid through the CARES Act, Response & Relief Act, and the American Rescue Plan were disbursed to individuals automatically, in the same manner as how they received tax refunds. Payments were disbursed primarily through electronic means, such as direct debit into a personal account, paper cheques, or prepaid debit cards.

**Private sector engagement**

*Engaging private health-care providers*

In India, Government directives were instituted to mobilise the private sector while setting standards, especially treatment and testing pricing. The Delhi Government asked private hospitals to reserve 80% of intensive care beds for patients with COVID-19. However, price caps were frequently exceeded and patients were left with high out-of-pocket costs.

In Mexico, an agreement with private hospitals that would join a single health strategy was made to offer care for conditions unrelated to COVID-19 to free up capacity within public hospitals for COVID-19 services by permitting the flow of Government monies to private hospitals.

In Singapore, funding was extended to Public Health Preparedness Clinics and Swab and Send Home clinics, which were private general practitioner clinics to support surveillance efforts and triage of patients. One-off payments were offered to cover set-up costs.

In South Africa, the Government agreed with the biggest three private health-care providers (ie, NetCare, Life Healthcare, and Mediclinic) to a fixed daily fee for uninsured critically ill patients with COVID-19 treated in critical care beds in private hospitals. The fee covered the cost of using the bed, paying the specialist team, and additional services such as pathology and radiology.

In the UK, a block-booking system of private hospital capacity and facilities was procured under contractual agreements between NHS Commissioners and NHS Trusts to deliver COVID-19 treatment and other services to increase response capacity, although it was broadly underused.

*Involvement of private insurance*

In Germany, the Federal Government liaised with the National Association for Social Health Insurance and the Association for Private Insurance to provide coverage for testing and treatment. Expenditure made by private health insurance on health services rose in 2020 by 1·7% to approximately €29 billion (US$32 billion).

In India, the Insurance Regulatory and Development Authority urged the private insurance industry to have some existing schemes cover COVID-19 costs while developing short-term suggested insurance packages including *Corona Kavach* and *Corona Rakshak* to cover hospitalisation costs on the basis of one-off premium payment. However, neither policy was offered for people older than 65 years, and the extent of their implementation is uncertain.

In Singapore, most private insurance companies automatically offered free COVID-19 coverage with no action required from the insured, even at the early stages of the pandemic without Government urging. Some companies also offered coverage for vaccine side-effects.

In Thailand, the Office of Insurance Commission (OIC) mandated life insurance to cover death due to COVID-19. The OIC mandated private insurance schemes to extend benefits coverage to treatment in field hospitals and hospitels (ie, hotels that collaborate with hospitals to provide COVID-19 care) and eventually cover home and community isolation.

In the USA, from March, 2020, to May, 2023, all co-payments were waived off for patients enrolled in Medicare and Medicaid, covering all treatment costs for COVID-19. COVID-19 vaccination will still be fully covered without co-payment until September, 2024.

**Expansion of coverage**

*Expansion of non-COVID-19 services coverage*

In Germany, social health insurance covered teleconsultation sessions for non-urgent non-COVID-19 care, and there were no limits to the number of sessions.

In Singapore, non-COVID-19-related telehealth consultation services that were traditionally not covered under Government subsidies were covered during the pandemic to reduce face-to-face consultation visits.

In Viet Nam, the cost of non-COVID-19-related medical examinations and treatment expenses when patients were in medical isolation was covered. The state budget covered the patient's co-payment and costs outside the scope of health insurance coverage.

*Expansion of COVID-19 services coverage*

In China, shortly after the first wave, the state issued a policy to include drugs and medical services for the treatment of COVID-19 as part of the payment range for the state medical insurance funds.

In Germany, the Second Act on the Protection of the Population was enacted to lessen financial consequences for ill health caused by COVID-19, including covering related services. Germany also covered the cost of European patients treated in the country if their home countries were unable to treat them due to capacity issues.

In Thailand, COVID-19 testing, treatment, and vaccination are provided free of charge to all Thai and non-Thai populations, including documented and undocumented migrant workers. The Ministry of Public Health covered the costs of the non-Thai population.

In the UK, all COVID-19 testing, treatment, and vaccination care were free at the point of use under the NHS until April, 2022, when free testing ended. Testing, treatment, and vaccination were also made free for all visitors to the UK and non-residents, including anyone living in the UK without permission (eg, undocumented migrants). No immigration checks were needed.

In the USA, the Health Resources and Services Administration COVID-19 Coverage Assistance Fund was implemented to cover the cost of administering COVID-19 vaccines to patients enrolled in health plans that do not cover vaccination fees or do so with patient cost-sharing during the early stages of the vaccination campaign.

Panel 2 **Country-level examples of maintaining expanded fiscal space for health and commitment towards universal health coverage**

**Maintaining expanded fiscal space**

*Increase and maintain revenue base*

In Indonesia, the Tobacco Products Excise Sharing Fund is a source of revenue from sin tax, which was used for health programmes such as the National Health Insurance Scheme and in 2021, for COVID-19 activities too.

In Singapore, the goods and services tax will be increased from 7% to 9% between 2022 and 2025, to draw more revenue to maintain the health sector and cope with the ageing population coupled with an increase in chronic disease burden.

In the UK, the Government planned an increase in National Insurance contributions for employees, employers, and self-employed from April, 2022, which was to be collected as a Health and Social Levy from April, 2023, to help cover the projected increase in health and social costs. This measure was abandoned in September, 2022, because the Government felt an increase in taxation would negatively affect economic growth, and the National Health Service continues to face substantial constraints, including from insufficient funding.

*Increase investment and expenditure on health*

In South Africa, the Government released the 2021 mid-term expenditure framework, which showed increased budget allocation for the National Health Insurance Program to strengthen the National Health Insurance Unit once it is created.

**Commitment towards universal health coverage**

*Political commitment*

In China, in 2021, the General Office of the State Council released its 14th Medical Security Plan (2021–25), stating that the country aims to establish a multi-tiered medical insurance system to provide basic medical security for all urban and rural residents and increase medical coverage for vulnerable individuals.

In Singapore, the Government regularly provides top-ups and subsidies for government-affiliated insurance coverage for older people to maintain longitudinal coverage while simultaneously rolling out more comprehensive schemes to cover more chronic conditions and enrolling more private sector providers in the face of an ageing population.

In South Africa, in June, 2023, lawmakers agreed on a National Health Insurance (NHI) bill that aims to pool private and public sector resources to maximise coverage by limiting the services offered by the private sector to those not covered by the NHI fund.

In Viet Nam, according to the Minister of Health, 90·85% of Viet Nam's population have been covered by Social Health Insurance in 2020. The Government has unveiled its goal to pursue universal health coverage, with the next target being 95% by 2025.

*Increase the reach of health-care coverage*

In Spain, from 2021, vulnerable groups such as low-income pensioners, moderately and severely disabled children, and households receiving child benefits no longer have to pay out of pocket for prescribed medicines.

In Thailand, in 2021, the Ministry of Labour assigned teams to inspect workplaces, and regularised any undocumented migrants found and put them into the health insurance scheme to ensure their coverage.

**Figure:**
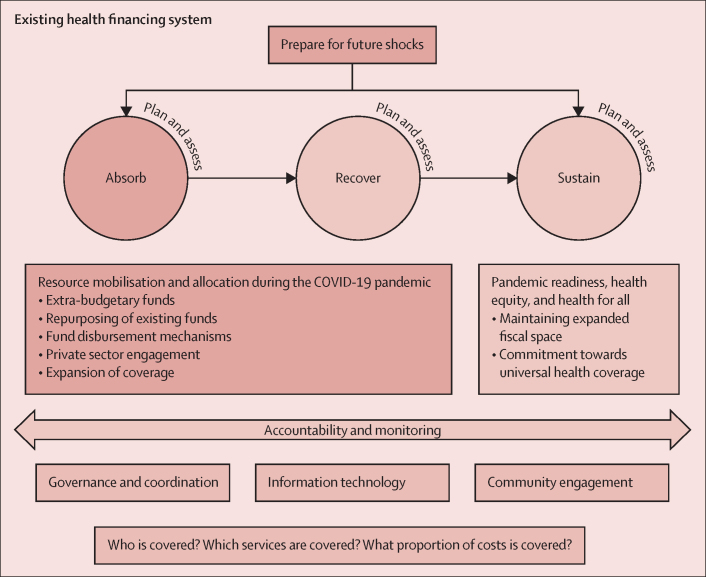
Framework for resilient health financing for health emergencies

Existing public financing mechanism systems need to remain malleable to rapidly deploy resources during a health emergency while planning and assessing measures adopted are carried out at every phase of the framework and modifications are made to prepare for any shocks that might arise. A health system needs to first absorb the shocks inflicted by the crisis by mobilising financial resources and allocating the funds swiftly to the targeted entities and personnel to serve as stopgap measures to reduce unnecessary morbidity and mortality. However, once an epidemic wave subsides or when a country anticipates a move towards endemicity, it can implement means to recover from the shock for both the population and the economy while continuing to plan and assess the evolving situation to mitigate any additional shocks to the system.

Extra-budgetary funds are accounts of transactions, often established in times of crisis to respond, with separate banking and institutional arrangements not included in the regular budget cycle. Extra-budgetary funds have been crucial during the early stages of the pandemic as countries required expeditious mobilisation of fiscal resources to fund national responses, encompassing procurement of medical necessities and offering of social and economic support packages. For example, advance cash transfers and even increased reimbursements for services were reported to expand health services, serve as financial safety nets in lieu of revenue loss, and protect health-care workers by providing resources to procure essential protective equipment.^25^ Traditional extra-budgetary funds, which are managed by state-owned or public entities, were used in countries with large national reserves, such as Brazil, Germany, and Singapore, through the mobilisation of treasury bonds or central reserves with approval from gate-keeping mechanisms, such as presidential authorisations under exceptional circumstances. By contrast, the establishment of privately managed funds was observed in Nigeria and South Africa, where private entities mobilised funds.^26^

Countries also availed support from international financial institutions, domestic banks, local donor initiatives, development partners, private sector, or bilateral government donations. For instance, Iran, South Africa, and Viet Nam received bilateral donations from other governments for their immediate national response plans, whereas countries such as Indonesia acquired loans from the World Bank and Asian Development Bank for social assistance programmes and vaccine financing. Indonesia simultaneously mobilised its central government reserves to complement these external loans.

Rapid mobilisation of existing funds catering to national responses can come from the reprioritisation of funds from low-priority to high-priority budgets through virements and repurposing. This approach was reported in most countries, where funds from non-pandemic budgets were reallocated to pandemic-related ones, and at times across sectors. India, Indonesia, and Mexico marshalled funds from budget lines not related to the health sector to pandemic budget lines, whereas Thailand appropriated part of its defence budget. Earmarking is another tool to ensure a pre-determined financial quantum is preserved. Germany, Mexico, Spain, and Thailand parameterised a set funding pool for the health system, whereas Brazil ringfenced funds to provide financial support to its population, to cushion the economic impact of the pandemic on households.

Efficient disbursement of funds for providers and populations is crucial during crises, whereby issuance of financial resources to intended personnel, including providers and the general population, was achieved through three key pillars of financial infrastructure: digital payment channels, digital identification system with wide population coverage, and data availability on individuals and enterprises linked to national identifications.^27^ This approach was observed in Brazil, India, Singapore, Thailand, and the USA. Specifically, general practitioners in Singapore could log onto a national health claims portal to submit claims for services rendered, facilitating faster digital disbursement. Furthermore, residents in Singapore have a personal bank account linked to the government, whereby vouchers, pay-outs, and support packages are directly credited. At the policy level, protocols were modified to quicken disbursement processes. In Spain, authorities cautiously relaxed administrative requirements for COVID-19 services, thereby removing previous authorisation from the Ministry of Finance and legal services to accelerate funding protocols while centralising its procurement efforts to expedite the logistical movement of supplies. Similarly, India streamlined bureaucratic requirements for purchasing medical supplies and authorised ministries at national and subnational levels to charge emergency spending to a special budget within a set limit early in the pandemic. India's Public Financial Management System, which leverages an existing database, was used for premium payment and relief disbursement.

The underlying principle is not to undermine fiscal controls but to create a system for managing priority disbursements and expediting authorisations. However, misappropriations of funds have been reported in some countries. In the UK, the government has faced heavy criticism for skipping usual quality, accountability, and transparency safeguards in procurement contracts, as well as allegations of cronyism. The procurement of personal protective equipment faced criticisms when some suppliers did not meet quality standards. Integrating technology that permits oversight and audit trails is foundational for the monitoring and accountability of movements and usage of funds. Accountability and monitoring must be upheld as over-riding principles across all phases.

To augment the public health sector, the private sector was engaged and reimbursed for its services. Broadly, the private sector had three functions in the countries analysed: it supported public health response, protected livelihoods, and adjusted health services.^28^ In most countries reviewed, the private sector was relatively disjointed from the public sector, and channelling government funds to the private sector was not systematic before the pandemic. In light of the pandemic, multiple governments instated legislation and mechanisms to route funds to these providers to expand the services available. These changes involved prospective agreements between purchaser and provider regarding terms and conditions of payments and type and volume of services during a defined period. Singapore provided private general practitioner funds for capital expenses to prepare to take on more patients and carry out infection and prevention control services in the community. The South African Government signed agreements with the country's three largest private health-care providers to regulate daily fees for critically ill patients with COVID-19 who were uninsured, covering the cost of the bed, medical team, and diagnostics. Such arrangements were not always effective. In the UK, government contracts with private hospitals were widely underused and were of questionable value for money. For example, very few patients with COVID-19 were treated in the 187 contracted private hospitals between March, 2020, and March, 2021, accounting for just 0·08% of COVID-19 bed days used nationally during that period.^29^

The private insurance sector also had a role in protecting the population from the medical costs derived from COVID-19 and non-COVID-19 health services. This approach was observed in some countries whereby distinct lines between the government and private insurance sector were drawn, such as Germany, Thailand, and the USA. Some governments, such as the Thai Government, mandated private insurers to cover the costs of COVID-19 services. In India, there were widespread reports of private insurance reimbursements falling short of the total bill for private care, fuelling the high catastrophic health costs observed during the pandemic.

Throughout the pandemic (and beyond), financial barriers to receiving non-COVID-19 and COVID-19 health services needed to be removed to ensure equitable access to these services when and where needed, while expanding all three dimensions of coverage policy—population coverage, services coverage, and removal of user fees—relieving service cost at points of care and covering the costs of non-traditional modes of health service delivery in the public facilities, including telemedicine. In most countries, governments quickly prioritised remote care, and a few even modified their providers’ payment systems to meet demand. However, as health-care workers are at the front lines, fair remuneration and protection is needed to retain them. Countries such as Germany and Singapore expanded coverage for all telehealth services, formerly not subsidised by the government, to free up hospital resources for more urgent care.

Expansion of financial protection also encourages care-seeking behaviour, thus supporting the COVID-19 response. All countries reviewed covered the costs of COVID-19 treatments and vaccinations using public financing, although covered services fell far short of demand in some instances, fuelling high catastrophic costs. Some countries also extended the entitlements to uninsured populations, although these measures were only temporary.^30^ Notably, most countries, at least in the initial stages of the pandemic, had also covered COVID-19-related expenses for non-residents, vulnerable groups, and undocumented migrants, as observed in Germany, Thailand, the UK, and a few states in the USA, promoting equitable access to health services for all.

The measures taken to safeguard lives and livelihoods during the pandemic need to be adapted to support the transition and accelerate post-pandemic recovery while sustaining public health gains. For a start, temporary measures which might have lost their utility in the sustain phase can be discontinued. A paradigm shift in health systems is also warranted to foster equitable, affordable access to quality health services. Unfortunately, the pandemic had increased the backlog of non-COVID-19-related cases; it also inevitably exposed the chronic under-investment in various parts of the health system such as the health-care workforce. As such, fiscal resources need to be set aside to invest in human resources among other building blocks of health systems as countries leave the pandemic behind.

Generating fiscal capacity for health is crucial for achieving UHC and the health-related SDGs. Fiscal capacity can be achieved by increasing domestic revenues, generating innovative revenue collection mechanisms, budget reprioritisation towards health, and efficiency gains.^31^ Strong governance is key to effectively implement these measures because it enables systems to uphold accountability and transparency across national and subnational levels. We observed that, in the third year of the pandemic, governments considered increasing taxation under some circumstances. Countries such as Indonesia and Singapore arranged to increase their goods and services taxes. The UK had planned to introduce a Health and Social Care Levy to expand revenue pools, but this measure was later abandoned, leaving the health service under substantial pressure including from funding constraints. Besides tax hikes, a few countries are also leveraging their sin tax on tobacco products; in Indonesia, this approach is used to fund its National Health Insurance scheme as its economy recovers. However, taxes on goods and services might exacerbate poverty in resource-constrained settings.

Ensuring the reach of health services to all, when needed, is fundamental to achieving UHC. The pandemic has also motivated governments to reaffirm their political commitment to UHC. China unveiled its National Medical Security Plan to offer a health security roadmap and South African lawmakers agreed in principle on a bill to maximise insurance coverage by rebalancing the services covered by private and public sectors. Governments also devised roadmaps to expand coverage compared with pre-pandemic levels, including enrolling non-residents and migrant groups into national insurance schemes, as reported in Thailand and Indonesia, and removing user charges for a wider range of medications for vulnerable groups, as reported in Spain.

Gravitating from a pandemic footing, countries need to carefully design the expansion of the access and coverage for health-care services, particularly for vulnerable groups, through an equity lens. Countries might have to find a way to systematically integrate a few necessary reforms that were temporarily adopted as well as implementing more expansive reforms to progress towards UHC. For instance, in the USA, 15 million people are estimated to be at risk of losing their health insurance coverage, provided during the pandemic, as the country officially ended the public health emergency on May 11, 2023.^30^ The country has adopted a decentralised transition out of the emergency and is facilitating enrolment and eligibility redetermination for coverage.

## Discussion

This Article provides an overview of fiscal adjustments made for health in response to the pandemic in 15 countries. Overall, countries absorbed shocks by leveraging existing financing policies while adapting financing systems to cater to the changing needs of the population and health systems and making resources available sufficiently and rapidly. Existing health financing systems need to remain malleable so resources can be rapidly deployed during an emergency while planning and assessments are carried out at every phase to prepare for any other accompanying shocks to the system.

Our framework is not meant to be deterministic; its components can be viewed as cyclical yet fluid, situated in a context of changing political landscapes, ageing populations, climate change, and the ongoing burden of the pandemic, to name a few dimensions. As depicted in the framework's cyclical nature, continuous planning and assessing of health financing mechanisms across stages is necessary to meet the evolving needs of the health system and prepare for future shocks. Centrally, political commitment to these fiscal changes requires fostering alliances at all levels of society and should transcend national boundaries to push for transformative changes to crisis response and health financing paradigm globally.

Adequate absorptive capacity is crucial, particularly during the early stages of an emergency, where countries require rapid funds and resources to surge health capacity quickly and retain health-care workers. Therefore, governments have had to mobilise existing resources and provide additional funding to health ministries and other purchasers of health services.^32^ Additional fund allocation was observed in countries with existing national reserves, although most countries relied heavily on borrowing. Countries in more precarious fiscal positions turned towards loans or receiving donations through many avenues from the outset. Although resources were commonly reallocated from other line items, this approach could worsen outcomes for other development indicators such as education and poverty, as also seen during the Ebola crisis in 2014, whereby education budgets were cut to make way for national responses to the crisis.^33^, ^34^

Most countries surged service capacity by recruiting retired medical staff, medical students, and migrant medics to meet the rising demand during the peak of the pandemic. The existing workforce was retained by providing bonus payments, such as incentive and welfare payments. However, with increased workload, the pandemic has stretched workers to their limit and caused excessive burnout, resulting in a shortage of health-care workers in most countries. This crucial health-system building block should be subjected to strategic investments and planning during non-emergency times—involving, for example, the provision of a conducive environment for training, commensurate financial reimbursements, the safeguard of mental health and wellbeing, and the minimisation of unnecessary bureaucracy and task-shifting where appropriate.^35^, ^36^

During the emergency, many countries engaged private sector providers to supply hospital care and testing services to complement public sector resources, with funding formalised through temporary contracting or pre-existing financing arrangements. To that end, numerous health systems adjusted provider payment methods to incentivise the provision of health services across the board, in both public and private settings, by deploying staff remunerated through salaries or by capitation in addition or in place of pay-for-performance or fee-for-services mechanisms.^25^ However, funds transferred to private providers need to be accounted for, which could be achieved by using accredited providers. Furthermore, some governments introduced regulatory provisions to reduce price exploitation, such as price ceilings for private providers, although these measures were often ineffective.^37^ In the absence of regulation and effective enforcement, corrupt practices might occur.^38^ Governments need to establish whether existing contractual arrangements are sufficient to scale up engagement with the private sector or whether adjustments or exclusive arrangements are needed in the emergency. Governments must also invest in robust regulation of private providers outside of health emergencies to mitigate against corruption, ensure quality of care, and enhance equitable access.

High out-of-pocket payments are a key deterrent for health-seeking behaviour, and have a profound impact on health equity. Therefore, the expansion of coverage for both crisis-related and non-crisis-related health services is paramount to maintaining population health in times of crisis. Most countries had removed user fees for COVID-19-related services for their residents, although the supply of free services could not match demand where public systems face chronic underfunding. A few countries also extended coverage to non-residents and undocumented migrants through newly established emergency funds or existing national disaster medical systems.^39^

To build back better, the positive gains derived from the adjustments made need to be sustained, and even more crucially, investments in health to protect population health need to prioritised, health systems strengthened, and health-care workers retained. During the COVID-19 pandemic, countries such as Germany, Thailand, and the UK covered COVID-19-related services for non-residents and undocumented migrants, whereas some states in India have gained momentum towards UHC by expanding health service coverage for vulnerable populations.^40^, ^41^ Temporarily expanded service and population coverage levels during the peak of the pandemic must be systematically integrated in the current financing mechanisms, so that the new beneficiaries are not at risk of losing their financial protection once the emergency officially ends. Furthermore, health services for non-COVID-19-related conditions must be progressively augmented to address the backlog created due to the prioritisation of outbreak management. Thus, financing systems responsive to evolving needs and changing fiscal space are warranted. Coverage expansion can be further achieved through regularisation efforts, such as the formalisation of the migrant workers’ health insurance scheme in Thailand, which extends health insurance coverage to a substantial number of documented migrants in the country.^42^, ^43^ Sustaining such expansions necessitates enlarging fiscal space, reinforcing the need for innovative financing, such as the usage of sin taxes to broaden the revenue base.

Transparency and monitoring are cornerstones in public financial management processes as they show how budgetary allocations are converted into expenditures. Although flexibility in public financial management and expedited approvals are necessary to rapidly respond to a crisis, it also increases opportunities for corruption across levels of purchasing and points of service delivery. Hence, strong governance of procurement processes for services and medical equipment is crucial, which also facilitates the ability to coordinate across subnational levels to mount a cohesive national response.^44^ In the absence of due diligence, health system responses are jeopardised because of shortages, delays, and misappropriations, and they might lead to wastage of fiscal resources. For example, Indonesia formed the National Committee for the COVID-19 Handling and National Economic Recovery, which oversees modifications to health financing strategies. Information technology tools can aid in this area too by forging interconnectivity across systems and maintaining fiscal transparency between issuers and recipients of the funds.^45^

Co-creating public health policies with end users is essential to maximise uptake. However, to garner the buy-in of all stakeholders, governments need to nurture trust and display fiscal transparency through community engagement. Lessons could be drawn from jurisdictions that have successfully experimented and formalised participatory budgeting (eg, in Brazil), whereby citizens can influence the mechanisms of resource allocation in their localities while ensuring transparency and accountability are upheld in the process.^46^, ^47^ In the context of public health emergencies, the rationale for modifications in health financing must be clearly conveyed to the general public, including when and how resources will be used. This aspect can be achieved by publishing independent audits of crisis-related spending and disbursement processes on government websites.

Our framework highlights the crucial elements explicated by the so-called UHC cube, which summarises the need for the three pillars (population covered, services covered, and financial protection) for universal coverage across stages (absorb, recover, and sustain).^48^ We argue that these pillars are not only integral for UHC but have proven even more crucial during the health crisis, to ensure the entire population, particularly vulnerable individuals, gained access to all COVID-19 services (eg, testing, treatment, isolation, and vaccine provision) and essential non-COVID-19 services, without financial repercussions.^5^ However, countries with higher scores on the UHC index as defined by WHO did not necessarily have lower infection and mortality rates. For instance, some countries with a high UHC index did not do as well as expected because of factors that are not directly linked to UHC.^49^ This outcome shows that multiple interacting factors exist, such as a country's pre-existing health financing system, fiscal flexibility combined with efficient and effective use of new funds, capacity to plan and assess on the basis of current fiscal position, and political economy, all of which need to be explored going forward.

Countries worldwide have expedited, modified, and implemented an array of measures, including legal, regulatory, policy, procurement, and service delivery innovations, to support their health systems in the past 3 years with additional funding. We have summarised a range of policy recommendations (panel 3) and additional closing remarks in the appendix (p 4). Going forward, it is a political choice to ensure that health systems are prepared when crises arrive and make progress towards UHC.

Panel 3 **Policy recommendations**

**Absorb and recover: expedite the flow of funds to the intended front line and populations using accountable and equitable mechanisms**

*Develop well coordinated national health financing strategies*

Clearly delineate the roles and responsibilities of all stakeholders from the government to health-care institutional levels to expedite decision making for planning, assessing, and modifying of policies and plans if needed during a health emergency.

*Map and address the service gaps*

Identify gaps in service delivery, particularly for services specific to the health crisis, and accordingly identify the services that require immediate prioritisation and associated public financial management mechanisms to fill these gaps while ensuring the running of services not related to the health crisis.

*Deploy public–private partnerships and strategic purchasing protocols*

Formulate pre-planned contractual agreements with private providers with accountability and quality assurance measures in place to complement public sector resources for health-care services delivery and manufacturing of medically essential products. Strengthen investment in the regulation of private health-care provision, particularly in countries with large or under-regulated private sectors.

*Strengthen digital infrastructure for digital health consultations, care integration, and cost coverage*

Leverage digital health tools for teleconsultation services for suitable health conditions (related or unrelated to the health crisis) while covering the cost of these services for the population. Adequate funding for well regulated providers to provide these services, which can be digitally disbursed, should also be adopted. Importantly, an interoperable information system is needed, which would integrate providers within and across levels of care and could also be equipped with monitoring mechanisms.

*Foster trust within the health system and with the population*

Governments must actively engage all stakeholders (eg, policy makers, health-care providers, and pharmaceutical and medical equipment companies) across the health system through fair and commensurate disbursement practices. The public must also be consulted and engaged through transparent communication of fiscal flows and explanation of how and when these public funds will be used.

*Place the most left behind first with an equity lens*

Vulnerable groups are most prone to suffering as a result of the direct effects of the health crisis as well as the unintended consequences brought about by public health measures. Health-care services must be made accessible to vulnerable individuals in a manner that does not incur financial hardship and is bound by human rights. Social and economic support measures must also be integrated into national response plans, with an equity focus to ensure that no population is left behind.

*Uphold transparency and accountability mechanisms*

Expeditious flows that in some crisis scenarios bypass stringent and bureaucratic auditing processes might expose health systems to mismanagement and misuse of funds. The channelling of financial resources to the population and providers, including health-care workers and businesses, must undergo scrutiny at multiple levels to ensure no conflict of interests occur, quality assurance processes are implemented, and that funds reach their intended targets.

**Sustain: use the health crisis as a window to progress the universal health coverage agenda and pandemic preparedness**

*Sustain and expand fiscal space for health*

Commit to using innovative and sustainable financing, such as increased tax mobilisation, supported by social health insurance contributions, and the usage of sin taxes on tobacco and sugar-sweetened beverages, to broaden the pool of funds available for health.

*Primer for political commitment*

Political changes are bound to happen, but political commitment and leadership for universal health coverage must remain impervious to political and economic cycles. Heads of states and other ministries such as ministries of finance will need to recognise that investing in equitable universal health coverage reforms will confer political and economic dividends in the long run. Political commitment for universal health coverage can also come in the form of embedding health into all economic growth and policy discussions.

*Set up a national reserve fund for health emergencies*

Set aside a portion of the revenue generated by the economy to go into a parameterised pool that is designated for health emergencies, which is separated from national reserves, and that can only be activated under exceptional circumstances through high-level authorisations.

*Sustained increase in expenditure for health*

Pooled public health financing needs to be enlarged for countries with low health expenditures and continually increased for others, such that health coverage for the population can expand, out-of-pocket expenditure can be reduced and replaced, and health systems can ensure minimal disruption to services during future emergencies.

*Global funding commitment for pandemic preparedness*

The mobilisation of fiscal resources should happen not only at the national level but also at international level because health emergencies transcend sovereign boundaries. The Financial Intermediary Fund for pandemic prevention, preparedness, and response offers a multilateral financing mechanism, which could be used by countries to address gaps in pandemic prevention, preparedness, and response and prepare for future health emergencies, honouring the notion that no one is safe until everyone is safe.

## Data sharing

Datasets of interview participants are de-identified and datasets from the desk reviews are organised at a country level. These datasets will be made available by the research team only upon reasonable request to the corresponding author from the time of publication.

## Declaration of interests

We declare no competing interests.
