# Supplementary material for: Health financing policies during the COVID-19 pandemic and implications for universal health care: a case study of 15 countries
Source: Lancet Glob Health. 2023 Nov 14;11(12):e1964–77. doi: 10.1016/S2214-109X(23)00448-5 (PMC10664823; doi:10.1016/S2214-109X(23)00448-5)
Supplement: Supplementary appendix [file mmc1.pdf]

THE LANCET
Global Health

## Supplementary appendix

This appendix formed part of the original submission and has been peer reviewed. We post it as supplied by the authors.

Supplement to: Foo CD, Verma M, Tan SY, et al. Health financing policies during the COVID-19 pandemic and implications for universal health care: a case study of 15 countries. *Lancet Glob Health* 2023; **11:** e1964–77.

**Appendix**

**Table of Contents**

Detailed Methodology ---------------------------------------------------------------------------------------------------------- Page 1
Search Strategy ------------------------------------------------------------------------------------------------------------------ Page 2
Topic Guide for Semi-structured In-depth Interviews ------------------------------------------------------------------ Page 3
Closing Remarks ------------------------------------------------------------------------------------------------------------------ Page 4

**Detailed Methodology**

Four-step process to data curation

This study employs a four-step process to explore the national health financing policy responses implemented during the pandemic. National University of Singapore Ethics committee approved this study (NUS-IRB-2021-172).

(i) Data gathering

We performed a search through PubMed, EMBASE, Scopus and Google Scholar between 19 December, 2021 and 30 October, 2022 and updated the search on 26 May 2023, to investigate how health systems financed their national responses and funnel fiscal resources for healthcare services during the COVID-19 pandemic by consolidating data from peer-reviewed papers (Appendix page 2). We also conducted documentary analysis of policy documents, technical reports, policy briefs, media releases as part of the data gathering process.

(ii) Filling the data gaps

We complemented the data we obtained with semi-structured in-depth interviews with key stakeholders, including policymakers, researchers, and medical practitioners in the fields of health systems and health financing from both private and public sectors. Participants were recruited through a combination of purposive and snowball sampling, whereby the sample size was determined through data saturation. All participants were recruited by sending invitation emails using their professional contact details available on public domains. 61 interviews were conducted with key stakeholders, with a preponderance from Asia from 8 September 2021 to 11 November 2021. The duration of the interview was between 30-90 minutes with a standard topic guide used to conduct the interviews (Appendix page 3). A total of 48 interviews were conducted via Zoom conference call, 10 were conducted via telephone calls and three were done face-to-face, whereby 50 were audio-recorded and field notes were collected for 10 participants who declined to be audio-recorded. All data were anonymized by using unique identifiers, transcribed verbatim and back translated if they were not conducted in English. It should be noted that the interviews were conducted to fill the gaps in our data as not all information was available on public domains and to validate collected data from the literature search.

(iii) Validating the data

The expanded study team comprised researchers with expertise in public health from 10 countries and in-depth knowledge of their own countries and others. We sent the consolidated data set to the expanded team for validation, highlighting any gaps requiring additional interviews.

(iv) Analysing the data using a conceptual framework

We adopted a combination of inductive and deductive approaches for this research. QSR NVIVO version 12 was used to carry out the thematic analysis (26). We adapted Barasa's framing of four key pillars to guide our analysis, which includes resource mobilization, resource allocation and purchasing, protecting people from financial hardship and sustainable progress towards UHC (23). During our thematic analysis, we uncovered additional domains, which were finalized after multiple rounds of iterative discussions with the expanded research team to gain consensus on the themes that capture the financing strategies implemented during the pandemic while situating resilience in health financing at the core of our framework (Figure 1). To implement effective responses to health emergencies, the health system must systematically *plan and assess* financing mechanisms but ensure sufficient flexibility to adapt existing health financing systems throughout and beyond the pandemic. We identified two key phases: 1) *Absorb and Recover*, where countries absorb the initial shock to health systems and establish mechanisms to recover from it, tackling the almost inevitable backlog of usual care (Panel 1) and 2) *Sustain*, ensuring that earlier progress towards sustainable health financing is maintained, and advancement and commitment to UHC remain priorities (Panel 2). Here we have merged *Absorb and Recover* phases as they involve similar processes.

**Search Strategy**

We searched the literature, including both government documents and peer-reviewed articles, using the search strategy stated below. Searches were conducted between December 2021 and May 2023.

Government documents, policy briefs and other technical reports were retrieved from websites of agencies, taskforce appointed for fund disbursement and management in all 15 countries. Similar searches were also conducted from international organization websites (eg., International Monetary Fund, World Bank, OECD) and academic literature databases (eg., PubMed, EMBASE and Scopus).

Key MeSH terms used were:

- Financing
- Global Health
- Pandemic / prevention & control
- Financial Management
- Financing Government
- Health Expenditure
- Fees and Charges
- Health Insurance
- Health budget

**Detailed search strategy for all databases is listed below.**

| **PubMed** | |
|---|---|
| #1 | ("Financing"[MeSH] OR "Financial Management"[MeSH] OR "Financing Government"[MeSH] OR "Health Expenditure"[MeSH] OR "Health Insurance"[MeSH] OR "User Fee"[MeSH] OR "Innovative Financing[MeSH]" OR "Payment Mechanism[MeSH]") AND ("Healthcare" OR "Health system" OR COVID*) |
| #2 | ("pandemic"[tiab] OR "COVID"[tiab]) AND ("health financing"[tiab] OR "financing"[tiab] OR "health budget"[tiab] OR "health insurance"[tiab] OR "public financial management"[tiab]) |
| #3 | #1 OR #2 |

| **EMBASE** | |
|---|---|
| #1 | ('health financing'/exp OR 'health expenditure'/exp OR 'financing government'/exp OR 'health insurance'/exp OR 'payment mechanism') AND ('healthcare'/exp OR 'health system'/exp OR 'COVID'/exp) |
| #2 | (pandemic:ti,ab,kw OR covid-19:ti,ab,kw) AND (health financing:ti,ab,kw OR financing:ti,ab,kw OR health budget:ti,ab,kw OR health insurance:ti,ab,kw OR public financial management:ti,ab,kw) |
| #3 | #1 OR #2 |

| **Scopus** |
|---|
| TITLE-ABS-KEY (financing OR financial management OR financing government OR health expenditure OR health insurance OR user fee OR innovative financing OR "payment AND mechanism" OR "health AND budget" OR "health AND insurance") AND ( "healthcare" OR "health AND system" OR covid-19 OR "pandemic") |

| **Google scholar** |
|---|
| We used the MeSH terms stated above and sieved through the first 10 pages of the searches due to the heterogeneity and amount of results yielded through this platform. |

**Topic guide for semi-structured in-depth interview**

**Introduction**

1. What does Universal Health Coverage (UHC) mean for your country?
2. How do you think UHC will affect pandemic response for your country?
3. How did the political commitment towards UHC evolve pre and post pandemic?
4. How do you define public finance management (PFM)?
5. How did financial resource allocation change in response to COVID-19?
6. What are the common ways countries modify their PFM strategies during a pandemic or an economic crisis?
7. What are some of the structural vulnerabilities in existing PFM architecture that might prohibit a country's capacity to prepare and respond to a pandemic?
8. How does a country decide when it is appropriate/ viable to modify PFM policies during a pandemic? Eg after a certain caseload, mortality, capacity of health infrastructure reached etc?
9. How important is social health insurance (SHI) during a pandemic? Are there instances whereby SHI did not augment a country's ability to weather a pandemic and why?
10. How do countries service their debt obligations during and post-pandemic?
11. How were the benefits package modified during the pandemic?
12. Which services were prioritized for the funding? Now that the country is moving from response to recovery how has this changed?
13. How has your country maintained accountability for how the resources are being spent?
14. How do international organizations such as the World Bank and Asian Development Bank (ADB) etc decide which countries to loan financial resources too? And how are these loans serviced by the countries?
15. During the pandemic how were the provider payment methods adjusted for COVID-19 and non-COVID-19 related health services?
16. To encourage healthcare seeking behavior, were user healthcare charges reduced or removed?
17. Please comment on the cost-sharing mechanism during this public health emergency.
18. How were pandemic related services tendered to public and private providers? In order to augment overall healthcare capacity, some countries had roped in the private healthcare sector to provide essential covid-19 related and non-related health services. How should the private sector be financed?
19. What role did independent purchasing agencies play in the decisions related to COVID-19 health response?
20. Please comment on the governance arrangements made since 2020 for purchasing.
21. Was the quality of health services delivered tracked? If so, how?
22. How were indirect costs due to loss of productivity caused by enforcement of non-pharmaceutical interventions (NPIs) covered?
23. What measures have been taken for the future as healthcare needs are likely to rise in a post-COVID-19 world?
24. What measures have been taken to provide for the vulnerable populations particularly those who may not have effective coverage and experienced dire consequences due to loss of employment? Have the steps been taken for undocumented migrant workers?
25. Where should the government invest more? Which services should be included in the benefits package? What changes should be made to PFM strategies post-pandemic or to prepare for the next health emergency?
26. How do countries raise domestic revenues rapidly for pandemic response?
27. How are financial aid packages disbursed to the population during a pandemic? E.g., direct cash transfer etc.? How can the process be made more efficient such that populations in need can receive financial aid on time and be of sufficient amount?
28. What are some exit strategies countries can deploy for their countercyclical policies?
29. Please share the lessons learnt for financing for future outbreaks.

**Closing remarks**

The COVID-19 pandemic represents two monumental shocks for the health system. Firstly, substantive new demands were placed upon the health system and there was a commensurate need to reallocate resources rapidly. Secondly, the global recession shrank fiscal space, affected employment rates, and possibly eroded financial support for UHC. These ramifications undermined health financing systems by lowering the income households have available to pay for health care services and, for systems financed by voluntary household contributions, by reducing the number of people contributing to risk pools and the number of people covered by health insurance. Further, a decline in economic activity will impact major sources of tax revenue, particularly value-added tax, while major aid contributors might look inwards and diminish foreign aid to lower-income countries.

This study offers insights into how countries with varying UHC indexes, with different health systems, modified their financing policies to fund their COVID-19 national responses. The findings offer policymakers a glimpse into trialled and tested strategies that augmented health system capacities at all levels, even as the world exits the emergency phase of the COVID-19 pandemic. However, we acknowledge pitfalls in the health financing strategies deployed. Policymakers can use the recommendations of this paper as guiding principles for re-engineering health financing processes and to argue a stronger case for UHC. The team acknowledges that the data is not representative of all countries and current practices are affected by a plethora of factors that fall outside the ambit of health systems, such as a country's political climate and other ongoing crises both foreign and domestic. Additionally, more longitudinal examination of the sustainability of fiscal policies to move countries towards UHC as the world slowly moves away from the COVID-19 pandemic is needed. The various facilitators and barriers to making health financing resilient during a health emergency is another area needing further research.

COVID-19 has demonstrated that UHC, strong public health systems and emergency preparedness are essential for protecting populations and economies. The pandemic has also underscored the perilous implications of excluding vulnerable groups unable to afford healthcare. Gaps in service coverage and delivery, and underfunded health systems are a major reason why an outbreak transcended into a pandemic of such scale, which has already cost the world more than USD 16 trillion (4). To quote WHO Director General Dr Tedros, "health is not a luxury, but a human right; not a cost, but an investment; not simply an outcome of development, but the foundation of social, economic and political stability and security." Investment in health will not just build greater resilience for future health shocks but also promote economic growth. It is estimated that better health could add USD 12 trillion to global GDP by 2040 (56). However, policies that expand fiscal space for health will need to be used to transform and make health systems more resilient by strengthening all building blocks including the healthcare workforce, especially as increasing numbers of healthcare workers leave the system during and post-pandemic.

Countries worldwide have expedited, modified and implemented an array of measures, including legal, regulatory, policy, procurement and service delivery innovations, to support their health systems in the last three years with additional funding. Going forward, it is a political choice to ensure that health systems are prepared when crises arrive and make progress towards UHC.
